# Accuracy of the Interpretation of Chest Radiographs for the Diagnosis of Paediatric Pneumonia

**DOI:** 10.1371/journal.pone.0106051

**Published:** 2014-08-22

**Authors:** Mohamed A. Elemraid, Michelle Muller, David A. Spencer, Stephen P. Rushton, Russell Gorton, Matthew F. Thomas, Katherine M. Eastham, Fiona Hampton, Andrew R. Gennery, Julia E. Clark, on behalf of the North East of England Paediatric Respiratory Infection Study Group

**Affiliations:** 1 Department of Paediatric Infectious Disease and Immunology, Newcastle upon Tyne Hospitals NHS Foundation Trust, Newcastle upon Tyne, United Kingdom; 2 Institute of Cellular Medicine, Newcastle University, Newcastle upon Tyne, United Kingdom; 3 Department of Radiology, Newcastle upon Tyne Hospitals NHS Foundation Trust, Newcastle upon Tyne, United Kingdom; 4 Department of Respiratory Paediatrics, Newcastle upon Tyne Hospitals NHS Foundation Trust, Newcastle upon Tyne, United Kingdom; 5 Biological, Clinical and Environmental Systems Modelling Group, School of Biology, Newcastle University, Newcastle upon Tyne, United Kingdom; 6 Regional Epidemiology Unit, Public Health England North East, Newcastle upon Tyne, United Kingdom; 7 Department of Paediatrics, Sunderland Royal Hospital, Sunderland, United Kingdom; 8 Department of Paediatrics, James Cook University Hospital, Middlesbrough, United Kingdom; 9 Department of Paediatric Infectious Disease, Royal Children’s Hospital, Brisbane, Queensland, Australia; Weill Medical College of Cornell University, United States of America

## Abstract

**Introduction:**

World Health Organization (WHO) radiological classification remains an important entry criterion in epidemiological studies of pneumonia in children. We report inter-observer variability in the interpretation of 169 chest radiographs in children suspected of having pneumonia.

**Methods:**

An 18-month prospective aetiological study of pneumonia was undertaken in Northern England. Chest radiographs were performed on eligible children aged ≤16 years with clinical features of pneumonia. The initial radiology report was compared with a subsequent assessment by a consultant cardiothoracic radiologist. Chest radiographic changes were categorised according to the WHO classification.

**Results:**

There was significant disagreement (22%) between the first and second reports (kappa = 0.70, *P*<0.001), notably in those aged <5 years (26%, kappa = 0.66, *P*<0.001). The most frequent sources of disagreement were the reporting of patchy and perihilar changes.

**Conclusion:**

This substantial inter-observer variability highlights the need for experts from different countries to create a consensus to review the radiological definition of pneumonia in children.

## Introduction

Chest radiograph is frequently performed when managing pneumonia in children [1], but usually does not affect the clinical outcome [2]. In epidemiological studies, the chest radiograph remains a major criterion in classifying pneumonia [3], [4]. However, variability in its interpretation for the diagnosis of pneumonia in children is a recognised problem [5]. It has been suggested that if radiologists follow the standardised World Health Organization (WHO) radiological definitions of pneumonia [3], this would allow more accurate comparative data in epidemiological studies for assessment of the impact of pneumococcal vaccination [4]. Broadly four categories are defined: “End-point consolidation”, “Other (non-end-point) infiltrate”, “Pleural effusion” and “No pneumonia” (Table 1).

**Table 1:** Summary of the WHO definitions of reporting chest radiographs in children with pneumonia [3].

1. “End-point consolidation”: a dense opacity that may be a fluffy consolidation of a portion or whole of a lobe or of the entire lung, often containing air bronchogram and sometimes associated with pleural effusion.
2. “Other (non-end-point) infiltrate”: a linear and patchy densities (interstitial infiltrate) in a lacy pattern involving both lungs, featuring peribronchial thickening and multiple areas of atelectasis with lung inflation is being normal to increased. It also includes minor patchy infiltrates that are not of sufficient magnitude to constitute primary end-point consolidation, and small areas of atelectasis which in children can be difficult to distinguish from consolidation.
3. “Pleural effusion”: this refers to the presence of fluid in the pleural space between the lung and chest wall. Mostly this will be seen at the costo-phrenic angle or as a layer of fluid adjacent to the lateral chest wall. This does not include fluid seen in the horizontal or oblique fissures. Pleural effusion is considered as primary end-point if it is in the lateral pleural space (and not just in the minor or oblique fissure) and is spatially associated with a pulmonary parenchymal infiltrate (including other infiltrate), or if the effusion obliterates enough the hemithorax to obscure an opacity.
4. “No pneumonia”: if there is no evidence of consolidation, infiltrate, or pleural effusion.

We conducted a study to explore the effect of the implementation of pneumococcal conjugate vaccine on the aetiology of childhood community-acquired pneumonia (CAP) [6]. Radiological findings were part of the study entry criteria. The aim of this analysis was to characterise inter-observer variability in the interpretation of chest radiographs for the diagnosis of pneumonia in children according to the WHO radiological classification [3].

## Methods

### Study Design and Participants

A prospective study to investigate the aetiology of CAP in children was undertaken from October 2009 to March 2011 in two teaching centres in North of England; the Newcastle Hospitals and South Tees Hospitals NHS Foundation Trusts [6]. Research teams of doctors and nurses led and ascertained the standardised diagnosis of pneumonia and the recruitment procedures across the two study centres. In the UK, children are assessed by a General Practitioner in primary care or accident and emergency team and then referred to a hospital-based paediatrician if secondary care is required.

Informed written consent was obtained from parents as well as assent from older children. Caldicott approval was granted and the study along with the informed consent procedures were ethically approved by the Newcastle and North Tyneside Research Ethics Committee (No: 08/H0906/105), and the Research Approval Board at South Tees Hospitals NHS Foundation Trust (No: 2008075).

Enrolled children aged ≤16 years who were presented to paediatric services with features suggestive of lower respiratory tract infection including any of fever, tachypnoea, dyspnoea, cough, respiratory distress and auscultatory chest crackles, with chest radiographic findings consistent with pneumonia as determined initially by the admitting paediatrician. Paediatricians were not asked to give specific radiological interpretations which were provided by radiologists. All children irrespective of the radiological findings received treatment for pneumonia according to the British Thoracic Society guidelines [7].

As this study was on the CAP aetiology, exclusions included clinical bronchiolitis or hospitalization in the preceding three weeks. Children with recent hospitalization were excluded in order to eliminate the potential risk of having hospital-acquired pneumonia rather than CAP. Children with underlying chronic chest diseases (such as cystic fibrosis) were also excluded to avoid any ambiguity in the interpretation of acute and chronic changes on chest radiographs.

### Laboratory Procedures

Microbiological and virological testing informed the aetiology of pneumonia which was previously published [6]. Identified pathogens were categorised as viral, bacterial or mixed viral-bacterial infections according to defined diagnostic criteria (Table 2) [6].

**Table 2:** Laboratory investigations and diagnostic criteria of likely causative pathogens of pneumonia.

Sample	Pathogen/antigen	Tests	Interpretation
Serum	Respiratory viruses	Complement fixation	Acute titre ≥1/128 or 4-foldrise between paired sera
	Atypical bacteria		
	*Mycoplasma*	IgM antibody	Positive
	Group A*Streptococcus*	Antistreptolysin O titre (IU/mL)	Acute 2-fold rise or 4-foldrise between paired sera
Blood	Bacteria	Culture	Growth
	*Streptococcus* *pneumoniae*	Real-time PCR	Positive
Nasopharyngealsecretions/sputum	Respiratoryviruses	Real-time PCR	Positive
Tracheobronchial secretions(collected via endotrachealtube or bronchoalveolar lavage)	Respiratoryviruses	Real-time PCR	Positive
	Bacteria	Culture/Real-time PCR	Growth/Positive
Pleural fluids	Bacteria	Culture	Growth
	Pneumococcalantigen	ELISA^1^	Positive
	*Streptococcus* *pneumoniae*	Real-time PCR	Positive

1 ELISA, enzyme-linked immunosorbent assay.

### Radiology

All chest radiographs were digitally taken, either with a flat panel detector or with a digital storage system. They were first reported by consultant radiologists locally as per routine clinical care and viewed electronically via the Picture Archiving and Communications System (PACS). There were uniform and regular quality assessments performed on the system performance including display characteristics. All reporters used similar workstations of radiological standards when reporting the chest radiographs. The location of the chest radiographic changes was documented by radiologists on every report and used for variability comparisons.

Using the full text written first reports, each radiograph was categorised into lobar (end-point consolidation), patchy, perihilar (non-end-point consolidation/infiltrate) or normal (no pneumonia) according to the WHO criteria [3], [4]. Effusion with fluid in the pleural space between the lung and chest wall was considered as primary end-point and classified simply as either present or absent [4]. This does not include fluid in the horizontal or oblique fissures as defined in the WHO radiological classification [4]. First reports were generated with the benefit of clinical information, a standard institutional requirement for routine reporting. All radiographs were reviewed by a second senior consultant cardiothoracic radiologist (MM) at the regional centre who was blinded to the first report. MM works primarily in paediatric radiology and regularly reports chest radiographs for children with cardio-respiratory diseases including pneumonia. Radiologists involved in performing the first and second reporting received the same training in radiology including the classification of radiological pneumonia.

A workshop including MAE, MM, DAS and JEC was carried out before the application of WHO criteria [3] on the first reports and performing the second reading in order to discuss and refine the potential definitions which could be a source of disagreement such as interstitial infiltrates of patchy or perihilar changes. There was a consensus agreement among the study team that if more than one radiographic change were reported, then in line with WHO recommendations the most significant one is reported [3]. The WHO criteria were prioritised according to the clinical significance, as follows: lobar (end-point consolidation) in favour of other changes (non-end-point infiltrates) if both were present [3]. There was no ambiguity on the wording of first reports that might cause confusion on categorization.

### Statistical Analysis

Inter-observer variability in the interpretation of chest radiographs was measured by the comparison of first reports with their second reading. Data analysis was performed using the PASW Statistics 19 program. The significance of inter-observer variability was assessed using fisher’s exact test because there were small values <5. Cohen’s kappa index (*k*) was calculated to measure the agreement between the first and second readers above that which would be expected by chance.

## Results

A total of 169 children were identified and treated for pneumonia and/or empyema (53% males, 73% aged <5 years, mean age 3.8±3.72 years, and age range from 0.05 to 16.7 years). Of those, 46 had chest radiograph reported as normal on the first reports, but on the second reading six (13%) had abnormal changes (i.e. false negative); four lobar and two patchy. All of the false negative cases received antibiotic treatment (median, 7 days), and none developed any complication. Fourteen (11.4%) were initially reported as having radiological changes, were reported as normal radiographs on the second review (i.e. false positive) (Table 3).

**Table 3:** Inter-observer variability and agreement in the chest radiographs reporting.

First reading		Second reading (gold standard)				Disagreement^1^
Radiographic changes	*n* (%)	Lobar	Patchy	Perihilar	Normal	*n* (%)
Lobar	48 (28.4)	47	1	0	0	1 (2.1)
Patchy	43 (25.4)	7	22	5	9	21 (48.8)
Perihilar	32 (19.0)	4	0	23	5	9 (28.1)
Normal	46 (27.2)	4	2	0	40	6 (13.0)
*Total*	169	62	25	28	54	37 (22.0)

1 Fisher’s exact test, *P*<0.001; Kappa = 0.70 (proportion of subjects on which readers would be expected to agree).

All radiologists agreed that all chest radiographs were suitable for interpretation. There was significant inter-observer variability in the interpretation of chest radiographs (*k* = 0.70, *P*<0.001), with patchy (48.8%) and perihilar (28.1%) changes being the main components of this variability (Table 3). Levels of disagreement were highest among children aged <5 years compared to those aged ≥5 years (26%, *k* = 0.66 versus 11%, *k* = 0.83, *P*<0.001). There was no disagreement on reporting lobar findings in the <5 years age group, disagreement was mainly related to patchy and perihilar changes.

Pleural effusion was present at first reading of the films in 10% (17/169) compared to 22% (37/169) on review. Variation in reporting of pleural effusion was 11.8% (*k* = 0.57, *P*<0.001). However, if the presence of a pleural effusion was reported in the first report there was no disagreement about this in the second report. In contrast 13.2% of pleural effusions were reported only on the second report and not in the first report.

## Discussion

We found substantial inter-observer variability in the interpretation of chest radiographs for the diagnosis of paediatric pneumonia. This has been recognized since radiology reporting was initiated in the middle of last century [8], [9], and continues despite the acceptance of the recommended WHO criteria for reporting chest radiographs of pneumonia in children [3], [4].

Yet, subtle radiographic changes can be difficult to recognise or interpret [10]. The initial interpretation of chest radiographs is usually performed by clinicians with the radiologists’ reports following later, often after the patient has been discharged from hospital [11]. Interpretation by clinicians could be biased by inadequate training in radiology and lack of clinical information may limit the accuracy of reporting by the radiologists [12]. For research purposes blinded interpretation of the chest radiograph may improve detection of subtle changes and differentiating normal biological variants [13]. Making clinical information available may reduce inter-observer variability but does not result in marked improvement in the overall accuracy [14].

This study shows that most inter-observer variability is related to the interpretation of patchy and perihilar changes, which need careful viewing and the availability of clinical information to facilitate their reading [15]. It is well recognised that abnormal chest radiographs may be interpreted as normal [15], but surprisingly four of the normal reports had lobar changes on review. Similarly, 13% had a previously undetected pleural effusion. The variation in reporting of chest radiographs for those aged <5 years confirms the particular challenge of making a radiological diagnosis of pneumonia in this age group [10], [16]. The findings in chest radiographs reported according to the WHO radiological classification of Pakistani children aged 2–59 months diagnosed with non-severe pneumonia showed normal films in 82% (1519/1848) and lobar consolidation in 26 children [17]. It is widely accepted in the literature that chest radiographs cannot reliably differentiate viral from bacterial aetiology of pneumonia [7], [18]. Therefore these variations on their interpretation do not significantly affect the clinical outcomes and management decisions of pneumonia in children [2], [7], [18].

Despite the specialized training in paediatric radiology and advanced technology, human error remains a likely factor [9]. The rate of false negative reports between the two interpretations of chest radiographs is a well-recognized problem [15]. This may jeopardize the results of epidemiological studies by underestimating the true burden of pneumococcal pneumonia [19]. Madhi and Klugman [19] raised a concern that radiologically-defined pneumonia according to the WHO criteria [3] may underestimate the actual effect of pneumococcal conjugate vaccine in preventing pneumococcal pneumonia by up to 63% when data from vaccine trial in South Africa were further analysed [19]. They suggested to include non-specific infiltrates that are associated with C-reactive protein level ≥40 mg/L when evaluating the vaccine impact [19].

In previous pneumococcal vaccine efficacy studies the radiographic evidence of pneumonia was observed in up to 34% of the enrolled children [20]. Despite the application of the WHO criteria [4], the concordance rate between two trained reviewers was only 48% (250/521) [21]. The degree of variability of reporting chest radiographs from the present study demonstrates that methodological differences are still a problem in the epidemiological studies of pneumonia in children. The WHO criteria still include a controversial term “infiltrate” which is no longer recommended in the Fleischner Society glossary of terms for thoracic imaging published by Hansell et al in 2008 [22], because it is non-specific [23], This may explain why there was highest discrepancy within this criterion.

### Considerations and Limitations

Our findings were limited by heterogeneity amongst a range of general and specialized radiologists involved in the first reporting, with only one radiologist performing second reporting. It has been recently shown among a group of 13 paediatricians and two radiologists that the main variability related to non-end-point changes [24]. Therefore the impact of heterogeneity on explaining this observed substantial reporting variability in our study is less likely. On the other hand the agreement between readers was improved when the WHO criteria [4] was modified to consider the presence of any lung infiltrate irrespective of its features as end-point pneumonia [25]. All of these reported findings highlight the importance to have defined radiological criteria of pneumonia that can be universally used in epidemiological studies and clinical practice.

### Clinical Implications

There is substantial inter-observer variability in the reporting of chest radiographs particularly in young children with pneumonia. These findings add to the recognized variability in the literature demonstrating that there may be a need for evaluation of the WHO classification of pneumonia in children to improve the validity and encourage widespread adoption of the criteria in the radiological diagnosis of this infection.

## References

[1] Esayag Y, Nikitin I, Bar-Ziv J, Cytter R, Hadas-Halpern I, et al (2010) Diagnostic value of chest radiographs in bedridden patients suspected of having pneumonia. Am J Med 123: 88 e81–85. 10.1016/j.amjmed.2009.09.012 20102999

[2] Swingler GH, Zwarenstein M (2008) Chest radiograph in acute respiratory infections. Cochrane Database Syst Rev: Art. No. CD001268. 10.1002/14651858.CD001268.pub3 18253988

[3] WHO (2001) Standardization of interpretation of chest radiographs for the diagnosis of pneumonia in children. World Health Organization Pneumonia Vaccine Trial Investigators Group, Geneva, WHO/V&B/01.35.

[4] Cherian T, Mulholland EK, Carlin JB, Ostensen H, Amin R, et al (2005) Standardized interpretation of paediatric chest radiographs for the diagnosis of pneumonia in epidemiological studies. Bull World Health Organ 83: 353–359. 15976876 PMC2626240

[5] Neuman MI, Lee EY, Bixby S, Diperna S, Hellinger J, et al (2012) Variability in the interpretation of chest radiographs for the diagnosis of pneumonia in children. J Hosp Med 7: 294–298. 22009855 10.1002/jhm.955

[6] Elemraid MA, Sails AD, Eltringham GJ, Perry JD, Rushton SP, et al (2013) Aetiology of paediatric pneumonia after the introduction of pneumococcal conjugate vaccine. Eur Respir J 42: 1595–1603. 23598951 10.1183/09031936.00199112 PMC3844138

[7] Harris M, Clark J, Coote N, Fletcher P, Harnden A, et al (2011) British Thoracic Society guidelines for the management of community acquired pneumonia in children: update 2011. Thorax 66 Suppl 2 ii1–23. 21903691 10.1136/thoraxjnl-2011-200598

[8] Garland LH (1959) Studies on the accuracy of diagnostic procedures. Am J Roentgenol Radium Ther Nucl Med 82: 25–38. 13661490

[9] Berlin L (2007) Accuracy of diagnostic procedures: has it improved over the past five decades? Am J Roentgenol 188: 1173–1178. 17449754 10.2214/AJR.06.1270

[10] Johnson J, Kline JA (2010) Intraobserver and interobserver agreement of the interpretation of pediatric chest radiographs. Emerg Radiol 17: 285–290. 20091078 10.1007/s10140-009-0854-2

[11] Klein EJ, Koenig M, Diekema DS, Winters W (1999) Discordant radiograph interpretation between emergency physicians and radiologists in a pediatric emergency department. Pediatr Emerg Care 15: 245–248. 10460076

[12] Loy CT, Irwig L (2004) Accuracy of diagnostic tests read with and without clinical information: a systematic review. JAMA 292: 1602–1609. 15467063 10.1001/jama.292.13.1602

[13] Swensson RG, Hessel SJ, Herman PG (1982) Radiographic interpretation with and without search: visual search aids the recognition of chest pathology. Invest Radiol 17: 145–151. 7076446 10.1097/00004424-198203000-00006

[14] Tudor GR, Finlay D, Taub N (1997) An assessment of inter-observer agreement and accuracy when reporting plain radiographs. Clin Radiol 52: 235–238. 9091261 10.1016/s0009-9260(97)80280-2

[15] Renfrew DL, Franken EA Jr, Berbaum KS, Weigelt FH, Abu-Yousef MM (1992) Error in radiology: classification and lessons in 182 cases presented at a problem case conference. Radiology 183: 145–150. 1549661 10.1148/radiology.183.1.1549661

[16] Stickler GB, Hoffman AD, Taylor WF (1984) Problems in the clinical and roentgenographic diagnosis of pneumonia in young children. Clin Pediatr (Phila) 23: 398–399. 6723188 10.1177/000992288402300707

[17] Hazir T, Nisar YB, Qazi SA, Khan SF, Raza M, et al (2006) Chest radiography in children aged 2–59 months diagnosed with non-severe pneumonia as defined by World Health Organization: descriptive multicentre study in Pakistan. BMJ 333: 629. 16923771 10.1136/bmj.38915.673322.80 PMC1570841

[18] Bradley JS, Byington CL, Shah SS, Alverson B, Carter ER, et al (2011) The management of community-acquired pneumonia in infants and children older than 3 months of age: clinical practice guidelines by the Pediatric Infectious Diseases Society and the Infectious Diseases Society of America. Clin Infect Dis 53: e25–76. 21880587 10.1093/cid/cir531 PMC7107838

[19] Madhi SA, Klugman KP (2007) World Health Organisation definition of “radiologically-confirmed pneumonia” may under-estimate the true public health value of conjugate pneumococcal vaccines. Vaccine 25: 2413–2419. 17005301 10.1016/j.vaccine.2006.09.010

[20] Puumalainen T, Quiambao B, Abucejo-Ladesma E, Lupisan S, Heiskanen-Kosma T, et al (2008) Clinical case review: a method to improve identification of true clinical and radiographic pneumonia in children meeting the World Health Organization definition for pneumonia. BMC Infect Dis 8: 95. 18644109 10.1186/1471-2334-8-95 PMC2492864

[21] WHO (2001) Model chapter for textbooks: IMCI, Integrated Management of Childhood Illness. World Health Organization, Geneva, WHO/FCH/CAH/01.01.

[22] Hansell DM, Bankier AA, MacMahon H, McLoud TC, Muller NL, et al (2008) Fleischner Society: glossary of terms for thoracic imaging. Radiology 246: 697–722. 18195376 10.1148/radiol.2462070712

[23] Patterson HS, Sponaugle DN (2005) Is infiltrate a useful term in the interpretation of chest radiographs? Physician survey results. Radiology 235: 5–8. 15798161 10.1148/radiol.2351020759

[24] Ben Shimol S, Dagan R, Givon-Lavi N, Tal A, Aviram M, et al (2012) Evaluation of the World Health Organization criteria for chest radiographs for pneumonia diagnosis in children. Eur J Pediatr 171: 369–374. 21870077 10.1007/s00431-011-1543-1

[25] Xavier-Souza G, Vilas-Boas AL, Fontoura MS, Araujo-Neto CA, Andrade SC, et al (2013) The inter-observer variation of chest radiograph reading in acute lower respiratory tract infection among children. Pediatr Pulmonol 48: 464–469. 22888091 10.1002/ppul.22644

